# LAT1 expression influences Paneth cell number and tumor development in Apc^Min/+^ mice

**DOI:** 10.1007/s00535-023-01960-5

**Published:** 2023-02-05

**Authors:** Yunlong Sui, Namiko Hoshi, Ryuichi Ohgaki, Lingling Kong, Ryutaro Yoshida, Norihiro Okamoto, Masato Kinoshita, Haruka Miyazaki, Yuna Ku, Eri Tokunaga, Yuki Ito, Daisuke Watanabe, Makoto Ooi, Masakazu Shinohara, Kengo Sasaki, Yoh Zen, Takenori Kotani, Takashi Matozaki, Zibin Tian, Yoshikatsu Kanai, Yuzo Kodama

**Affiliations:** 1 Division of Gastroenterology, Department of Internal Medicine, Kobe University Graduate School of Medicine, Hyogo, 650-0017 Japan; 2 Department of Bio-system Pharmacology, Graduate School of Medicine, Osaka University, Osaka, 565-0871 Japan; 3 Integrated Frontier Research for Medical Science Division, Institute for Open and Transdisciplinary Research Initiatives (OTRI), Osaka University, Osaka, 565-0871 Japan; 4 Division of Molecular Epidemiology, Kobe University Graduate School of Medicine, Hyogo, 650-0017 Japan; 5 The Integrated Center for Mass Spectrometry, Kobe University Graduate School of Medicine, Hyogo, 650-0017 Japan; 6 Graduate School of Science, Technology and Innovation, Kobe University, Hyogo, 657-8501 Japan; 7 Institute of Liver Studies, King’s College Hospital, London, SE5 9RS UK; 8 Division of Molecular and Cellular Signaling, Department of Biochemistry and Molecular Biology, Kobe University Graduate School of Medicine, Hyogo, 650-0017 Japan; 9 Department of Gastroenterology, The Affiliated Hospital of Qingdao University, Qingdao, 266000 China

**Keywords:** L-type amino acid transporter 1, Paneth cells, Cancer, mTORC1, Wnt3

## Abstract

**Background:**

Amino acid transporters play an important role in supplying nutrition to cells and are associated with cell proliferation. L-type amino acid transporter 1 (LAT1) is highly expressed in many types of cancers and promotes tumor growth; however, how LAT1 affects tumor development is not fully understood.

**Methods:**

To investigate the role of LAT1 in intestinal tumorigenesis, mice carrying LAT1 floxed alleles that also expressed Cre recombinase from the promoter of gene encoding Villin were crossed to an Apc^Min/+^ background (LAT1^fl/fl^; vil-cre; Apc^Min/+^), which were subject to analysis; organoids derived from those mice were also analyzed.

**Results:**

This study showed that LAT1 was constitutively expressed in normal crypt base cells, and its conditional deletion in the intestinal epithelium resulted in fewer Paneth cells. LAT1 deletion reduced tumor size and number in the small intestine of Apc^Min/+^ mice. Organoids derived from LAT1-deleted Apc^Min/+^ intestinal crypts displayed fewer spherical organoids with reduced Wnt/β-catenin target gene expression, suggesting a low tumor-initiation capacity. Wnt3 expression was decreased in the absence of LAT1 in the intestinal epithelium, suggesting that loss of Paneth cells due to LAT1 deficiency reduced the risk of tumor initiation by decreasing Wnt3 production.

**Conclusions:**

LAT1 affects intestinal tumor development in a cell-extrinsic manner through reduced Wnt3 expression in Paneth cells. Our findings may partly explain how nutrient availability can affect the risk of tumor development in the intestines.

**Supplementary Information:**

The online version contains supplementary material available at 10.1007/s00535-023-01960-5.

## Introduction

Epidemiological studies show that the incidence of intestinal tumors in both small and large intestines is increasing and that increased intake of red or processed meat alters the risk of intestinal tumor development [1–3]; however, the mechanism by which those nutrients contribute to the incidence of intestinal tumors is not well understood. Amino acids, which are metabolized from those foods [4], are not only required to build body structures, such as muscles but also act as signaling molecules in cells to modulate signaling pathways, such as the mTOR pathway, which participate in body regulation [5, 6]. Although essential to physiological function, excessive levels of amino acids can contribute to prevalent diseases, such as heart failure, diabetes, and cancer [7, 8]. Therefore, it is important to elucidate the link between amino acids and these diseases.

L-type amino acid transporter 1 (LAT1; encoded by *Slc7a5*) is an important member of the sodium-independent amino acid transport system L, which forms a complex with glycoprotein 4F2hc to import most neutral amino acids, such as leucine, valine, and phenylalanine [9, 10]. The mTOR pathway is activated when these amino acids are transported by LAT1 [11]. LAT1 is only expressed in some normal tissues, such as endothelial cells and placenta [12, 13], and absent in many normal tissues, such as the pancreas, breast, and lung; moreover, it is highly expressed in tumors originating from these organs [14–16]. LAT1 is also upregulated in human intestinal tumors and associated with accelerated tumor cell proliferation [17]. However, whether LAT1 plays any other role in intestinal tumorigenesis is not well understood.

Adenomatous polyposis coli (*APC*) is a tumor-suppressor gene, and its mutation causes aberrant activation of the Wnt pathway that promotes tumor growth. Familial adenomatous polyposis (FAP), which causes multiple adenomas in the intestines and induces small intestinal and colonic cancers in patients, develops due to *APC* mutation [18]. Using a murine model of intestinal tumor Apc^Min/+^ mice, we aimed to investigate the link between LAT1 expression and intestinal tumorigenesis; to this end, we generated Apc^Min/+^ mice with LAT1 deficiency in the intestinal epithelium. Our study could provide valuable insights regarding the relationship between nutrition and intestinal tumor incidence.

## Methods

### Animals and diets

Mice were bred under specific pathogen-free conditions in the Animal Facility at Kobe University Graduate School of Medicine. Villin-Cre and Apc^Min/+^ mice (strain #004586 and #002020, respectively) were purchased from the Jackson Laboratory (Bar Harbor, ME, USA). LAT1^fl/fl^ mice were generated as previously described [19], and LAT1^fl/fl^; vil-cre; Apc^Min/+^ and LAT1^fl/fl^; Apc^Min/+^ mice were generated by breeding these strains. All mice were of a C57BL/6J background, and both female and male mice were used in all experiments. All animal experiments were approved by the Institutional Animal Care and Use Committee of Kobe University (approval number: P190307).

### Tumor count

At 15 weeks old, the small intestine and colon were excised and cut longitudinally. The tumor number and size were recorded in a blinded manner by the same researcher throughout the study.

### Histology

The terminal part of the small intestinal tissue was harvested, fixed in formalin (#133-10311; FUJIFILM Wako), and embedded in paraffin. Using the sections of hematoxylin–eosin staining, three intact crypts were randomly selected from each sample, and Paneth cells were manually counted under a microscope (400× magnification) by a researcher who was blinded to the mouse genotypes. For immunohistochemistry, the following primary antibodies were used: Ki67 (1:100; #12202; Cell Signaling) and LAT1 (provided by Osaka University [19]). The secondary antibody #K4003(Dako) was used for Ki67 staining. For LAT1 staining, the ABCHRP Kit Peroxidase (#pk6101; Vector Laboratories) was used. ImageJ software (National Institutes of Health, Bethesda, MD, USA) was used for the analysis of Ki67-positive cells by two researchers who were blinded to the mouse genotypes.

### Detection of apoptosis

Apoptotic cells were stained using the TUNEL Assay Kit-HRP-DAB (#ab206386; Abcam). The experimental procedure was performed according to the manufacturer’s instructions. The number of apoptotic cells in three different high-power fields (HPFs, 400× magnification) was counted individually by two researchers who were blinded to the mouse genotype of the specimens. The calculated average number/HPF values were used as data.

### RNA extraction and real-time PCR

Intestinal tumor and normal tissues were harvested and placed in RNAlater (#AM7021; Thermo Fisher Scientific). Total RNA was extracted using TRIzol^®^ reagent (#15596018; Thermo Fisher Scientific). For organoid RNA extraction, the RNeasy Mini kit (#74104; QIAGEN) was used according to the manufacturer’s protocol. The extracted RNA was reverse-transcribed into cDNA using a High-Capacity cDNA Reverse Transcription Kit^®^ (#4374967; Applied Biosystems) according to the manufacturer’s protocol. Real-time PCR analysis was performed using SYBR Green (#4367659; Applied Biosystems) on an ABI 7500 real-time PCR system (Applied Biosystems). The relative expression levels of the target genes were standardized to hypoxanthine–guanine phosphoribosyltransferase (HPRT) expression. The primers used are listed in Table 1.

**Table 1:** Primers used for real-time PCR in this study

Gene	Forward (5′–3′)	Reverse (5′–3′)
*Defa4*	CCAGGGGAAGATGACCAGGCTG	TGCAGCGACGATTTCTACAAAGGC
*Defa5*	AGGCTGATCCTATCCACAAAACAG	TGAAGAGCAGACCCTTCTTGGC
*Lyz1*	GCCAAGGTCTACAATCGTTGTGAGTTG	CAGTCAGCCAGCTTGACACCACG
*Ccnd1*	CTCCGTATCTTACTTCAAGTGCG	CTTCTCGGCAGTCAAGGGAA
*Ddit3*	CTCGCTCTCCAGATTCCAGTC	CTTCATGCGTTGCTTCCCA
*Wnt3*	TAAAGTGTAAATGCCACGGGTT	CGGAGGCACTGTCGTACTTG
*Wnt6*	CGGAGACGATGTGGACTT	GGAACCCGAAAGCCCATG
*Wnt9b*	ACCTGAAGCAGTGTGACCTAC	GCTCCTGCCTGAACTGGAA
*Axin2*	GGACTGGGGAGCCTAAAGGT	AAGGAGGGACTCCATCTACGC
*Ctnnb1*	CCCAGTCCTTCACGCAAGAG	CATCTAGCGTCTCAGGGAACA
*C-Myc*	GAACTTACAATCTGCGAGCC	AGTCGAGGTCATAGTTCCTG
*Slc7a5*	TGATGCGTCCAACCTGCAGC	ATCCTCCGTAGGCGAAGAGG
*Il-6*	GACAAAGCCAGAGTCCTTCAGAGAGATACAG	TTGGATGGTCTTGGTCCTTAGCCAC
*Il-22*	GGTGACGACCAGAACATCCA	GACGTTAGCTTCTCACTTTCCT
*Tnf-α*	AAAATTCGAGTGACAAGCCTGTAG	CCCTTGAAGAGAACCTGGGAGTAG
*Hprt*	GTTGGATACAGGCCAGACTTTGTTG	CCAGTTTCACTAATGACACAAACG

### Western blotting

Proteins were extracted in RIPA buffer, and the concentration was determined using a BCA Protein Assay kit (#23227; Thermo Fisher Scientific). Proteins were run in SDS-PAGE gel (8% for p-S6K1, 10% for β-actin and Wnt3, 12% for CHOP, 15% for p-4E-BP1, cyclin D1, and caspase-3). The following primary antibodies were used: p-S6K1 (1:1000; #9234; Cell Signaling), p-4E-BP1 (1:1000; #9455; Cell Signaling), cyclin D1 (1:1000; #2978; Cell Signaling), caspase-3 (1:1000; #9662; Cell Signaling), CHOP (1:1000, #2895; Cell Signaling), Wnt3 (1:1000, #ab172612; Abcam), and β-actin (1:2000; #8457; Cell Signaling). Incubation with primary antibodies was followed by incubation with anti-rabbit IgG (H + L) secondary antibody (1:5000; #31458; Thermo Fisher Scientific) or m-IgGκ BP-HRP (1:1000; #SC-516102; Santa). Western blot chemiluminescent signals were captured with an ImageQuant LAS 4000 mini imager (Fujifilm). The results were derived using ImageQuant TL software (GE Healthcare), and band intensity was measured using ImageJ (NIH).

### Organoid culture and analysis

The terminal ileum tissue was excised, maintained in ice-cold phosphate-buffered saline (PBS), cut into 0.5 cm long pieces, and incubated in PBS containing 2.5 mM EDTA on a rotator at 4 °C for 30 min. The tissue pieces were transferred to 45 mL PBS containing 10% FBS, vortexed and filtered; crypts were collected and counted. Two hundred crypts per well were plated in Matrigel (#356231; Corning), and 250 μL basic culture medium was added using 48-well plates. The basic culture medium contained advanced DMEM/F12 (#12634-010; Gibco), 100 U/mL penicillin/streptomycin (#26253-84; Nacalai Tesque), 10 mM HEPES (#15630080; Gibco), 1 × Glutamax (#35,050–061; Gibco), 1× B27 supplement (#17504044; Thermo Fisher Scientific), 1× N2 supplement (#17502048; Life Technologies), 100 ng/mL Noggin (#PEP-250-38; Peprotech), 50 ng/mL mEGF (#PMG8041; Thermo Fisher Scientific), 1.25 mM *N*-acetylcysteine (#A9165-5G; Sigma-Aldrich), and 10% R-spondin1 conditioned medium. For the Wnt3a supplementation experiments, Afamin/Wnt3a CM (#J2-001; JSR Life Sciences) was added to the basic culture to obtain a final concentration of 10% Wnt3a. Organoids were counted in a blinded manner on day 5. Five spherical (Apc^Min/+^) organoids were randomly selected, and the diameter and calculated average were used as the diameter/spherical organoid data.

### Statistical analysis

Data are presented as the mean ± standard error of the mean (SEM). Prism 7 (GraphPad Software Inc.) was used for all the analyses. When two conditions were compared, an unpaired two-tailed Student’s *t* test was used. When more than two conditions were compared, a one-way analysis of variance (ANOVA) followed by Bonferroni’s multiple-comparisons test or Kruskal–Wallis test followed by Dunn’s multiple-comparisons test was applied. A value of *p* < 0.05 was considered statistically significant.

## Results

### LAT1 was constitutively expressed in intestinal crypts: conditional deletion of LAT1 led to fewer Paneth cells in the normal small intestine at the steady state

First, to confirm the expression of LAT1 in adenoma and intestinal cancer, we performed immunohistochemistry for LAT1 using clinically obtained human samples. As expected, LAT1 was highly expressed in both colonic adenomas and adenocarcinomas but expressed at low levels in normal tissues (Fig. S1). Next, we generated intestinal epithelium-specific LAT1-deleted animals by crossing mice carrying LAT1 floxed allele(s) with a mouse line expressing Cre recombinase under the villin promoter (hereafter vil-cre) to generate LAT1^fl/fl^; vil-cre mice [20]. As it is known that whole-body deletion of LAT1 is embryonically lethal [21], we wanted to ensure that the conditional deletion of LAT1 in the intestinal epithelium would not lead to lethality. The LAT1^fl/fl^; vil-cre mice were born in Mendelian ratios (Fig. S2) with no observable gross growth retardation based on body weight. Deletion efficacy was calculated to be approximately 90% via real-time PCR and confirmed using western blotting (Fig. S3).

**Fig. 1:**
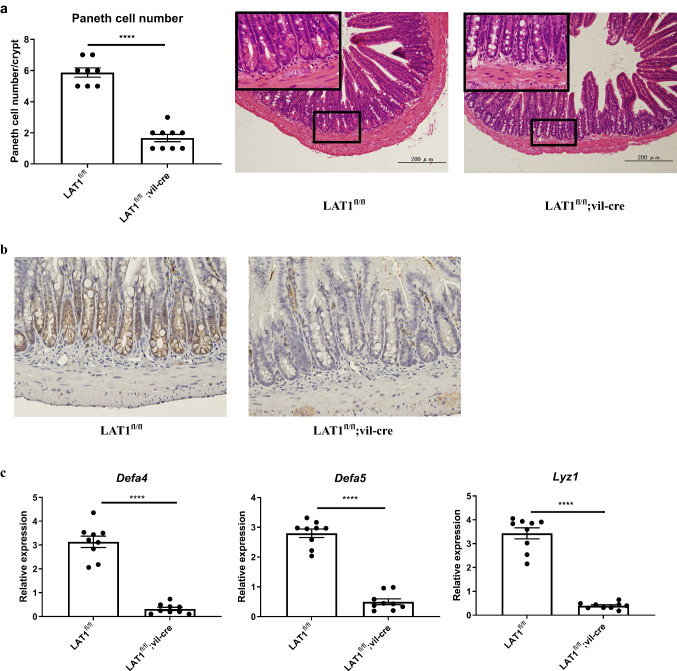
LAT1 was expressed in small intestinal crypts; conditional deletion of LAT1 led to fewer Paneth cells in the normal epithelium. **a** Paneth cells/crypts were counted in the small intestines of LAT1^fl/fl^ control (*n* = 8) and LAT1^fl/fl^; vil-cre mice (*n* = 9) mice. Representative images of hematoxylin and eosin staining (×200 and ×1000 magnification [inset]). **b** Immunohistochemical staining of LAT1 in the small intestine. (×400 magnification). **c** Relative antimicrobial peptide gene expression levels in the small intestinal tissues were measured using real-time PCR. LAT1^fl/fl^ (*n* = 9), LAT1.^fl/fl^; vil-cre (*n* = 9); Error bars indicate the mean ± standard error of the mean (SEM); statistical analysis was performed using an unpaired two-tailed Student’s *t* test. ****p < 0.0001

To determine whether LAT1 deletion affected intestinal structure, histological analysis was performed. The colon tissue was comparable between LAT1^fl/fl^; vil-cre and LAT1^fl/fl^ mice; however, the number of Paneth cells, a type of epithelial cells seen in the small intestinal crypts, was substantially reduced in LAT1^fl/fl^; vil-cre mice compared with that in LAT1^fl/fl^ mice (Fig. 1a). We thought that this phenotype should not be observed if LAT1 is only expressed in the intestinal tumor cells; thus, we conducted immunohistochemistry for LAT1 on normal intestinal tissue sections. LAT1-positive cells were confirmed in the crypt base cells in both the small intestine and colon (Fig. 1b, Fig. S4), suggesting that LAT1 expression is critical to the Paneth cell number. To objectively confirm the reduced number of Paneth cells, we analyzed the expression levels of antimicrobial peptide encoding genes *Defa4, Defa5,* and* Lyz1*, which are known to be expressed in Paneth cells. As expected, the expression levels of all these genes were substantially reduced in LAT1^fl/fl^; vil-cre mice compared with those in LAT1^fl/fl^ mice (Fig. 1c). These results suggest that LAT1 is expressed in the crypts at the steady state and affects the development of Paneth cells in the small intestine.

### Conditional deletion of LAT1 in the intestinal epithelium reduced tumor number and size in the small intestine but not in the colon of Apc^Min/+^ mice

**Fig. 2:**
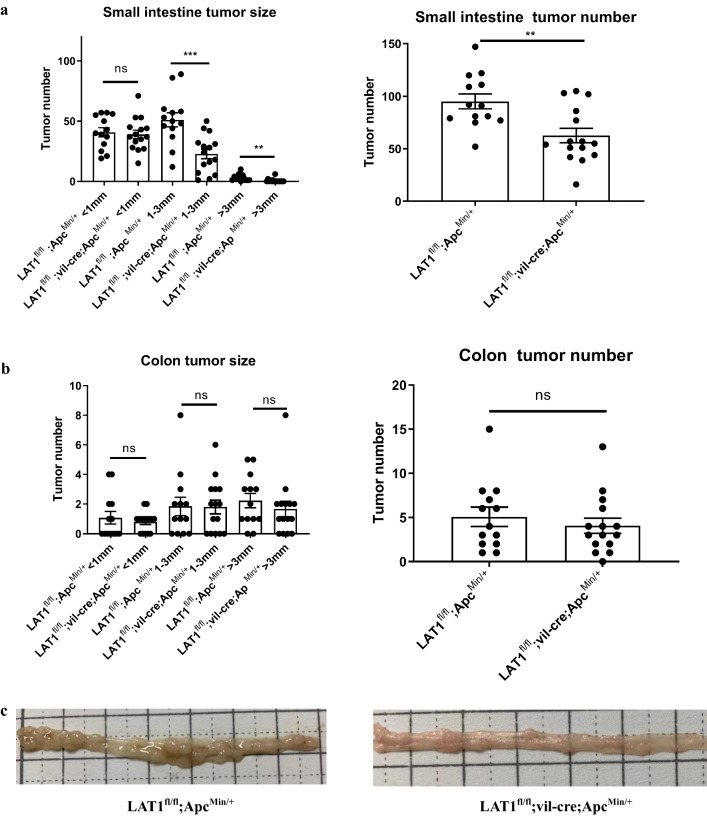
Conditional deletion of LAT1 in the intestinal epithelium reduced tumor number and size in the small intestine but not in the colon of Apc^Min/+^ mice. **a** Tumor number and size were counted and measured in the small intestine and **b** the colon at 15 weeks old. LAT1^fl/fl^; vil-cre; Apc^Min/+^ (*n* = 15); LAT1^fl/fl^; Apc^Min/+^ (*n* = 13). **c** Representative macroscopic pictures of the terminal ileum. Error bars indicate mean ± SEM; statistical analysis was performed using an unpaired two-tailed Student’s *t* test. **p < 0.01, ***p < 0.001. *ns* no significance, *p* > 0.05

**Fig. 3:**
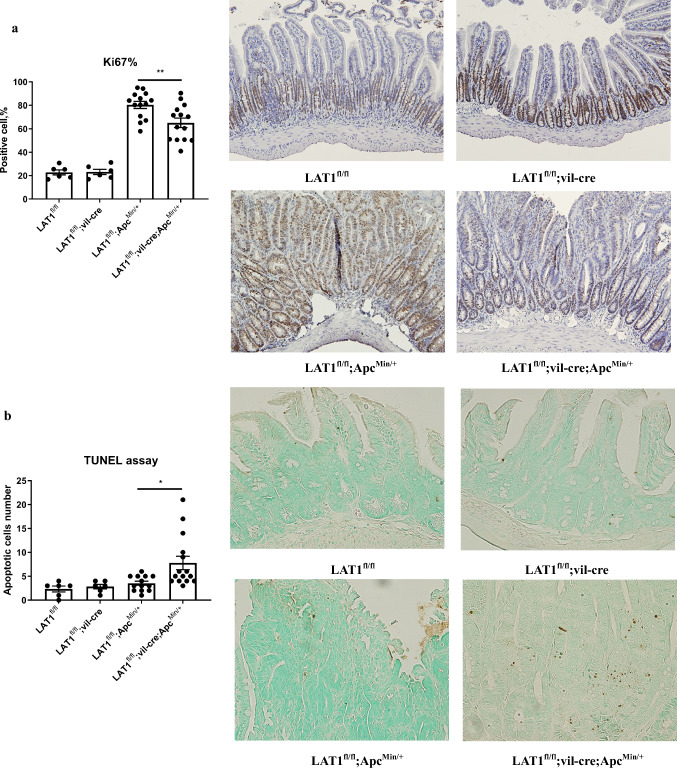
Cell proliferation was reduced, while the number of apoptotic cells was increased in LAT1^fl/fl^; vil-cre; Apc^Min/+^ tumors. **a** Ki67 staining was performed on small intestinal tissues, and representative pictures are shown (× 200). The percentage of positive cells was analyzed using ImageJ software. LAT1^fl/fl^ (*n* = 6), LAT1^fl/fl^; vil-cre mice (*n* = 6); LAT1^fl/fl^; Apc^Min/+^ (*n* = 14), LAT1^fl/fl^; vil-cre; Apc^Min/+^ mice (*n* = 14). Error bars indicate mean ± SEM; statistical analysis was performed using one-way ANOVA followed by Bonferroni’s multiple-comparisons test. **b** TUNEL staining was performed to detect apoptotic cells on small intestinal tissues, and representative pictures are shown. The total positive cell numbers of three different high-power fields (HPF: ×400 magnification) from each section were counted and shown as the average number/HPF. LAT1^fl/fl^ (*n* = 6), LAT1^fl/fl^; vil-cre; (*n* = 6); LAT1^fl/fl^; Apc^Min/+^ (*n* = 13), LAT1^fl/fl^; vil-cre; Apc^Min/+^ mice (*n* = 15). Error bars indicate mean ± SEM; statistical analysis was performed using Kruskal–Wallis test followed by Dunn’s multiple-comparisons test. *p < 0.05, **p < 0.01

To investigate the role of LAT1 in intestinal tumor development, LAT1^fl/fl^; vil-cre mice were crossed with Apc^Min/+^ mice, and tumor formation was analyzed. LAT1 staining confirmed that the entire tumor was LAT1-positive in LAT1^fl/fl^; Apc^Min/+^ mice and LAT1-negative in LAT1^fl/fl^; vil-cre; Apc^Min/+^ mice, which might suggest that LAT1-positive cells in the crypt base include stem cells because Apc ^Min/+^ tumors are shown to develop from stem cells [22] (Fig. S5). LAT1^fl/fl^; vil-cre; Apc^Min/+^ mice exhibited fewer and smaller tumors in the small intestine than LAT1^fl/fl^; Apc^Min/+^ mice. However, no differences were observed in the number or size of colonic tumors (Fig. 2a–c). To investigate the mechanism by which LAT1 deletion results in smaller and fewer tumors in the small intestine, we performed Ki67 and TUNEL staining to analyze the status of cell proliferation and apoptosis in the tumors, respectively. LAT1^fl/fl^; vil-cre; Apc^Min/+^ tumor tissues exhibited a lower number of Ki67-positive cells (Fig. 3a) and a higher number of apoptotic cells (Fig. 3b). These findings indicate that the conditional deletion of LAT1 influences tumor cell proliferation and causes tumor cells to become apoptotic to critically affect tumor development in the small intestine of Apc^Min/+^ mice.

### Conditional deletion of LAT1 suppressed the mTORC1 pathway in tumors

**Fig. 4:**
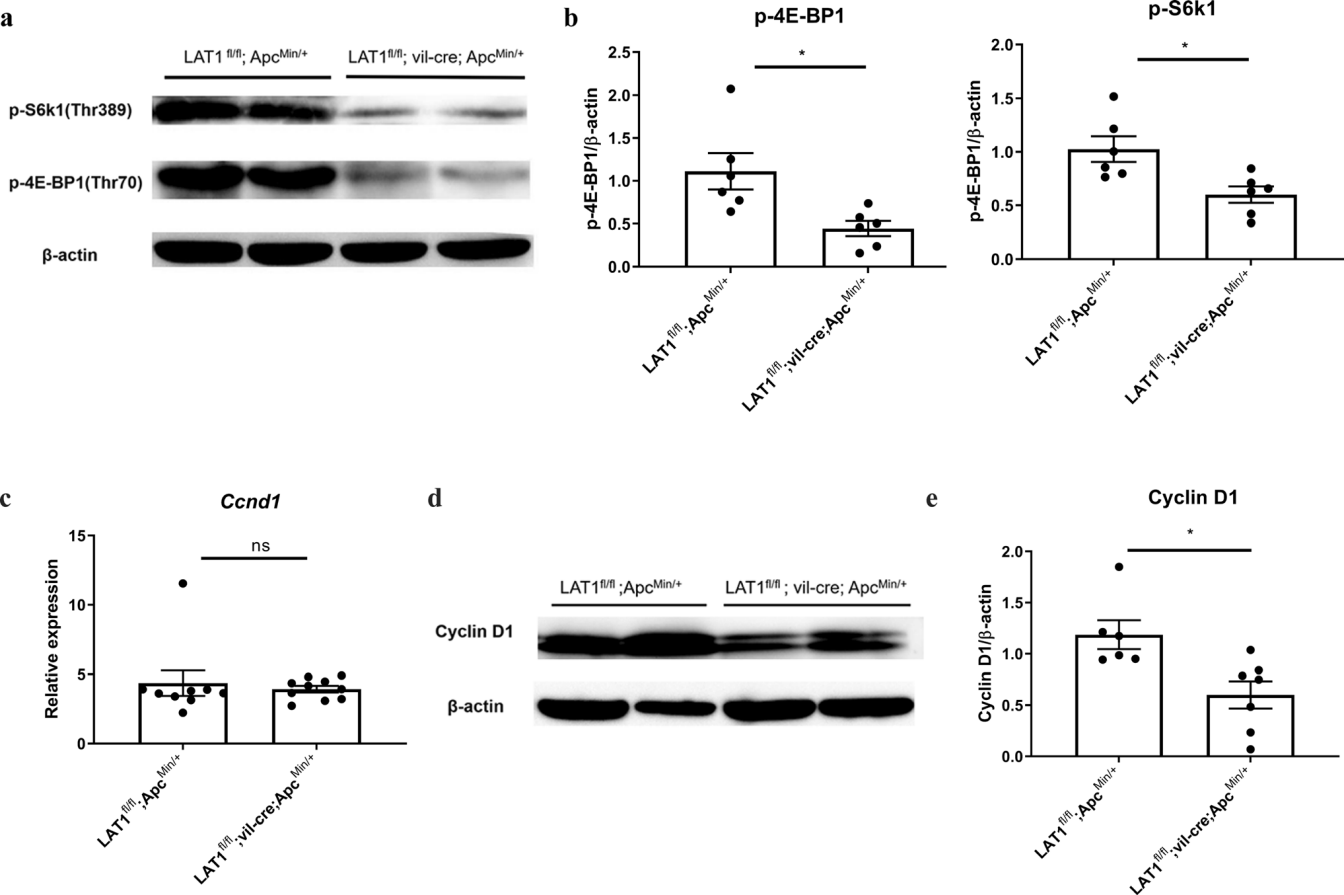
Conditional deletion of LAT1 suppressed the mTORC1 pathway in tumors. **a** Comparison of mTORC1 activation was performed using western blotting of p-S6k1 and p-4E-BP1 in intestinal tumor tissue. **b** The band intensity was measured using ImageJ software. LAT1^fl/fl^; Apc^Min/+^ (*n* = 6), LAT1^fl/fl^; vil-cre; Apc^Min/+^ mice (*n* = 6). **c** Gene expression of *Ccnd1* (encodes Cyclin D1) was measured using real-time PCR. LAT1^fl/fl^; LAT1^fl/fl^; Apc^Min/+^ (*n* = 9), LAT1^fl/fl^; vil-cre; Apc^Min/+^ mice (*n* = 10). **d** The expression of Cyclin D1 at the translational level was examined using western blotting, **e** and the band intensity was measured using ImageJ. LAT1^fl/fl^; Apc^Min/+^ (*n* = 6), LAT1^fl/fl^; vil-cre; Apc.^Min/+^ mice (*n* = 7). Error bars indicate mean ± SEM; statistical analysis was performed using an unpaired two-tailed Student’s *t* test. *p < 0.05, *ns* no significance, *p* > 0.05

The mammalian/mechanistic target of the rapamycin complex 1 (mTORC1) pathway plays an important role in cell proliferation and growth [23, 24] and is also shown to be activated downstream of LAT1 [19]. To determine whether mTORC1 activation was inhibited in LAT1^fl/fl^; vil-cre; Apc^Min/+^ tumors, we assessed the phosphorylation status of eukaryotic translation initiation factor 4E-binding protein 1 (4E-BP1) and ribosomal protein S6 kinase 1 (S6K1). As expected, the phosphorylation of 4E-BP1 and S6K1 was reduced in LAT1^fl/fl^; vil-cre; Apc^Min/+^ tumors (Fig. 4a, b). The cyclin D1 gene (*Ccnd1*), which is highly expressed in many tumors [25] and is a target of the Wnt pathway, is also regulated by the mTORC1 pathway to promote cell division at the translational level [26, 27]. The mTORC1 pathway is a master regulator of mRNA translation [28, 29]. We found that the expression of* Ccnd1* mRNA levels did not differ between LAT1^fl/fl^; Apc^Min/+^ and LAT1^fl/fl^; vil-cre; Apc^Min/+^ tumors, as measured by real-time PCR, but were reduced at the protein level in LAT1^fl/fl^; vil-cre; Apc^Min/+^ mice (Fig. 4c-e). This confirms that LAT1 deficiency indeed modifies mTORC1 activation, not at the transcriptional level of the target genes but at the translational level, as previously described [30]. These results suggest that the conditional deletion of LAT1 inhibits tumor growth by impairing cell proliferation by reducing activation of the mTORC1 pathway.

### Conditional deletion of LAT1 caused tumor apoptosis in vivo

**Fig. 5:**
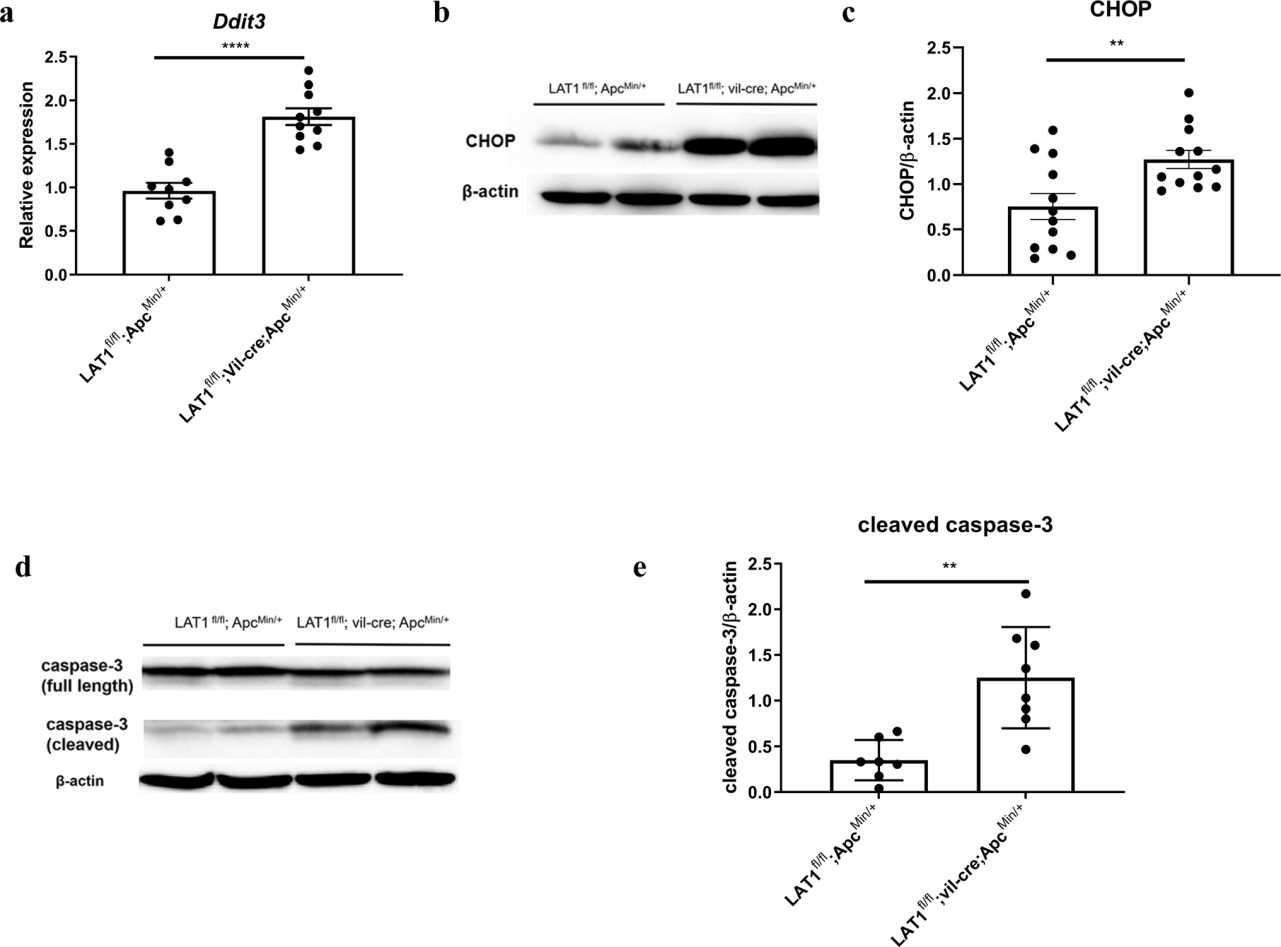
Conditional deletion of LAT1 caused tumor apoptosis in vivo. **a**–**c** The expression levels of *Ddit3* were measured using real-time PCR and western blot in the tumors. For real-time PCR, LAT1^fl/fl^; Apc^Min/+^ (*n* = 9), LAT1^fl/fl^; vil-cre; Apc^Min/+^ mice (*n* = 10). For western blot, LAT1^fl/fl^; Apc^Min/+^ (*n* = 12), LAT1^fl/fl^; vil-cre; Apc^Min/+^ mice (*n* = 12). **d**, **e** Western blotting results and analysis of caspase-3 are shown in each group. LAT1^fl/fl^; Apc^Min/+^ (*n* = 7), LAT1^fl/fl^; vil-cre; Apc^Min/+^ mice (*n* = 8). Error bars indicate mean ± SEM; statistical analysis was performed using an unpaired two-tailed Student’s *t* test. **p < 0.01, ****p < 0.0001

The endoplasmic reticulum (ER) stress response, including ATF4/CHOP pathway activation, is triggered by extracellular environmental challenges, such as nutrient deprivation, including glucose or amino acids, and can result in apoptosis [31, 32]. As LAT1 deficiency could reduce the supplementation of amino acids to the tumor cells [33], we hypothesized that the ER stress response was induced in LAT1^fl/fl^; vil-cre; Apc^Min/+^ tumors. Indeed, the expression levels *Ddit3* (encoding CHOP) were substantially increased in the LAT1^fl/fl^; vil-cre; Apc^Min/+^ tumors compared with those in LAT1-sufficient tumors (Fig. 5a–c). Caspase-3 is recognized as an apoptosis executioner; its activation can be measured by its cleavage [34]. We confirmed that the cleavage of caspase-3 was considerably increased in LAT1^fl/fl^; vil-cre; Apc^Min/+^ tumors compared with that in LAT1^fl/fl^; Apc^Min/+^ tumors (Fig. 5d, e). Notably, an increased number of apoptotic cells was observed in the tumors but not in the normal tissues of the LAT1^fl/fl^; vil-cre mice (Fig. 3b). These results suggest that LAT1 deficiency increases apoptosis in tumor tissues but not in normal crypts.

To determine whether other factors could be involved in the phenotype of reduced tumor burden in the LAT1^fl/fl^; vil-cre; Apc^Min/+^ mice, inflammation and gut microbiota components are analyzed since both can affect tumor growth [35–38]. Some inflammatory cytokine gene expressions were tested via real-time PCR; however, no difference was detected between LAT1^fl/fl^; Apc^Min/+^ and LAT1^fl/fl^; vil-cre; Apc^Min/+^ tumors (Fig. S6a). Paneth cells play an important role in controlling the microbiota [39], and some components such as Lactobacillus and Bifidobacterium are reported to inhibit tumor development in Apc^Min/+^ mice [38]. Although Paneth cell reduction in the LAT1^fl/fl^; vil-cre mice was observed, the 16S rRNA sequences using small intestinal contents showed no dramatic difference between LAT1^fl/fl^ and LAT1^fl/fl^; vil-cre mice at the steady state (Fig. S6b, c). Another possibility was that LAT1 deficiency in the intestinal epithelium affected the amino acid levels in the circulation and feeding tumor cells. However, we confirmed that the amino acid concentration was not altered (Fig. S6d). These results suggested that LAT1 deletion affects tumorigenesis not through the alteration of the inflammatory status, gut microbiota, and amino acid levels in the circulation in our setting.

### Organoids derived from LAT1-deleted tumors displayed fewer and smaller spherical organoids

**Fig. 6:**
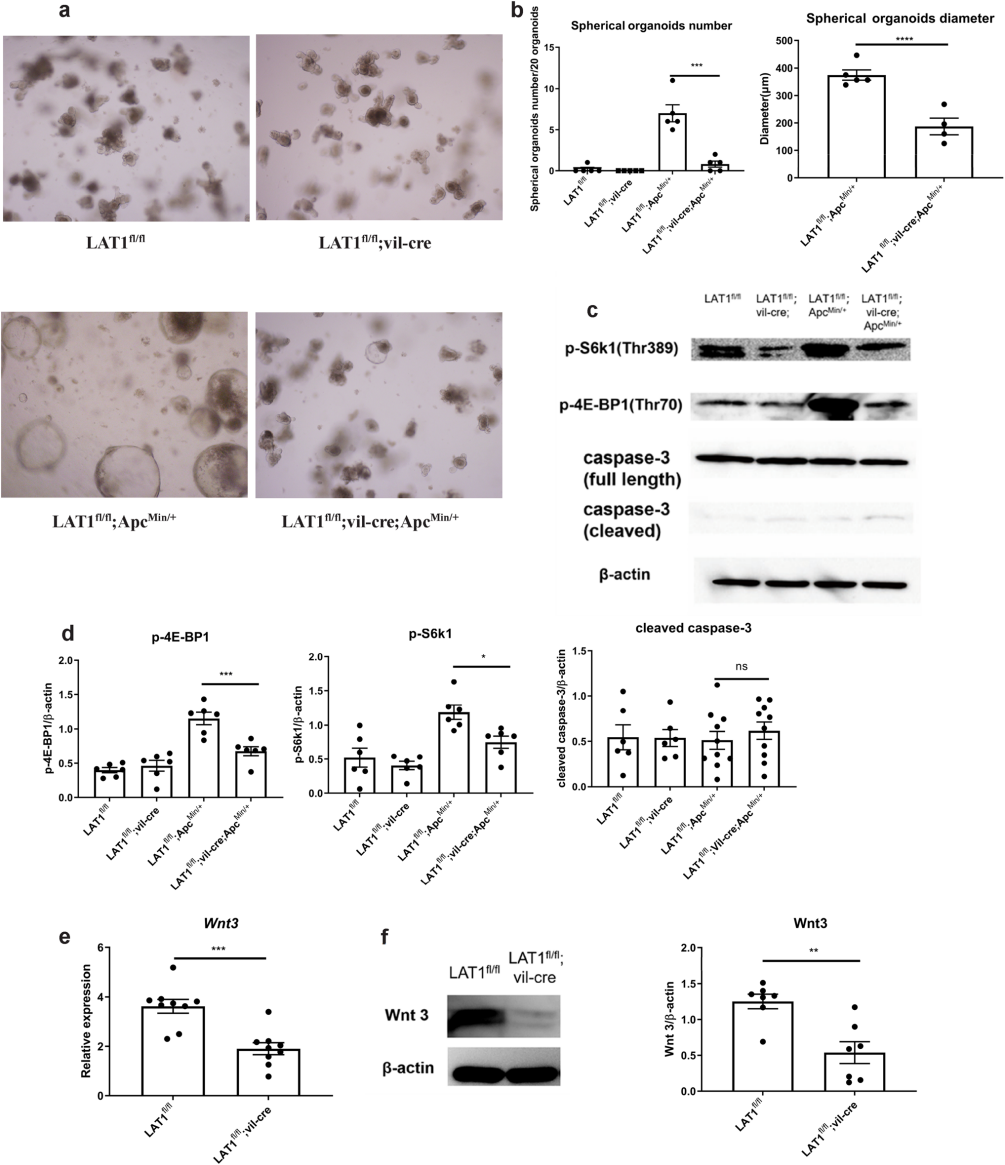

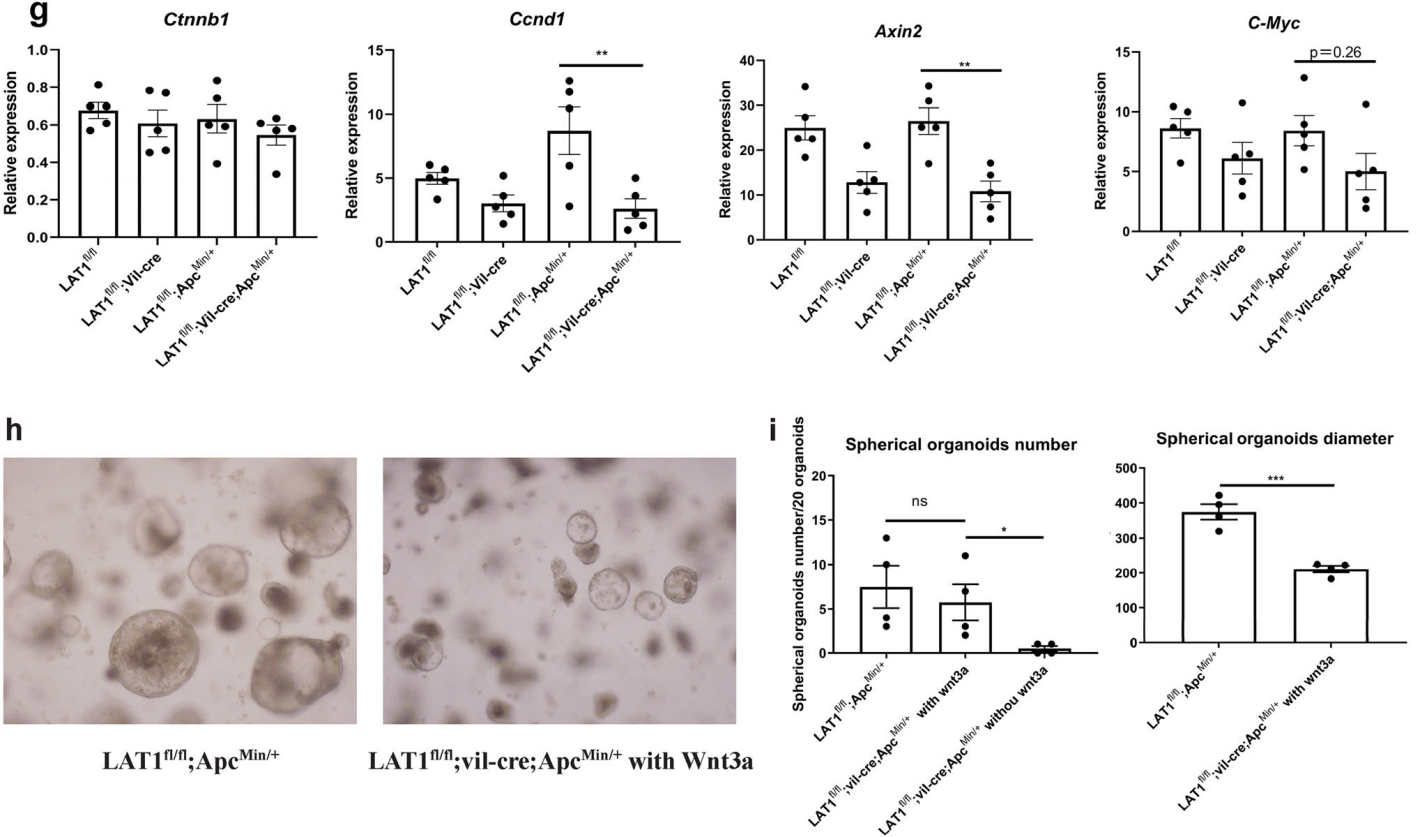
Organoids derived from LAT1-deleted tumors displayed fewer and smaller spherical organoids. **a** Intestinal organoids were cultured using crypts isolated from the small intestines of each genotype. Representative images are shown (×40 magnification). **b** The number and size of Apc ^Min/+^ (spherical) organoids are recorded in each group. LAT1^fl/fl^ (*n* = 5), LAT1^fl/fl^; vil-cre; (*n* = 5); LAT1^fl/fl^; Apc^Min/+^ (*n* = 5), LAT1^fl/fl^; vil-cre; Apc^Min/+^ (*n* = 5). **c**, **d** Western blotting results and analysis of p-S6k1, p-4E-BP1, and cleaved caspase-3 band intensity were measured using ImageJ. LAT1^fl/fl^ (*n* = 6), LAT1^fl/fl^; vil-cre (*n* = 6); LAT1^fl/fl^; Apc^Min/+^ (*n* = 6), LAT1^fl/fl^; vil-cre; Apc^Min/+^ (*n* = 6). For caspase-3, LAT1^fl/fl^; Apc^Min/+^ (*n* = 10), LAT1^fl/fl^; vil-cre; Apc^Min/+^ (*n* = 10). **e**, **f** The expression of *Wnt3* was measured using real-time PCR, and the protein levels using western blotting in each group. For real-time PCR, LAT1^fl/fl^ (*n* = 9), LAT1^fl/fl^; vil-cre (*n* = 9); For western blotting, LAT1^fl/fl^ (*n* = 7), LAT1^fl/fl^; vil-cre (*n* = 7). **g** The expression levels of target genes of the Wnt/β-catenin pathway (*Ccnd1, Axin2, C-Myc*) and non-target gene *Ctnnb1* were measured using real-time PCR. LAT1^fl/fl^ (*n* = 5), LAT1^fl/fl^; vil-cre (*n* = 5), LAT1^fl/fl^; Apc^Min/+^ (*n* = 5), LAT1^fl/fl^; vil-cre; Apc^Min/+^ (*n* = 5). **h** Crypts isolated from LAT1^fl/fl^; vil-cre; Apc^Min/+^ small intestine were cultured with supplementation of 10% Wnt3a in the medium. (**i**) The number and size of Apc ^Min/+^ organoids were counted and measured in each group. LAT1^fl/fl^; Apc^Min/+^ (*n* = 4), LAT1^fl/fl^; vil-cre; Apc^Min/+^ (*n* = 4). Error bars indicate mean ± SEM; statistical analysis was performed using an unpaired two-tailed Student’s t-test (two conditions) or one-way ANOVA followed by Bonferroni’s multiple-comparisons test (more than two conditions). *p < 0.05, **p < 0.01, ***p < 0.001, ****p < 0.0001, *ns* no significance, *p* > 0.05

In vivo analysis showed increased apoptosis in LAT1^fl/fl^; vil-cre; Apc^Min/+^ tumors; however, whether the absence of LAT1 directly induced apoptosis in the cell has not been conclusive. Therefore, LAT1 knockdown by siRNA experiments was carried out using the colon cancer cell lines SW480 and LoVo containing the Apc mutation; however, LAT1 knockdown did not increase caspase-3 cleavage (Fig. S7). For further analysis, we utilized cultured small intestinal organoids. The crypts from LAT1^fl/fl^; vil-cre; Apc^Min/+^ mice formed fewer and smaller spherical organoids than LAT1^fl/fl^; Apc^Min/+^ crypts, which matched the in vivo phenotypes (Fig. 6a, b). LAT1-deficient organoids showed reduced p-S6K1 and p-4E-BP1 levels recapitulated the in vivo phenotype; however, no clear promotion of caspase-3 cleavage was confirmed. These data suggest that LAT1 deletion can directly affect mTORC1 activation and suppress tumor cell proliferation; however, the promotion of apoptosis seems to be an indirect effect only detected in vivo (Fig. 6c, d).

To gain further insight into the mechanisms by which the tumor number could be reduced in the small intestine of LAT1^fl/fl^; vil-cre; Apc^Min/+^ mice, we formulated two hypotheses: the same number of tumors was initiated, but slow tumor growth or increased apoptosis resulted in undetectably small tumors; or tumor initiation was reduced in the LAT1^fl/fl^; vil-cre; Apc^Min/+^ mice. We assumed that the latter hypothesis was more likely because it was clear that there was substantially fewer spherical organoid formations derived from the LAT1^fl/fl^; vil-cre; Apc^Min/+^ small intestine (Fig. 6a). Thus, we further speculate that Paneth cells are involved in this process. In addition to antimicrobial peptides, Paneth cells produce Notch ligands, epidermal growth factor, Wnt3, to form the intestinal stem cell niche [40, 41]. Lgr5^+^ stem cells are considered the origin of Apc-deficient tumors [22] and overactivation of the Wnt pathway is key for tumor formation. To this end, we first investigated whether LAT1 deletion could reduce the number of stem cells. We observed Lgr5-positive cells using RNA in situ hybridization (RNAScope) and immunohistochemical staining of Olfm4 [42] (Fig. S8). However, the positive cell numbers were similar between LAT1-sufficient and -deficient intestinal crypts, suggesting that the reduction in tumors was not due to fewer stem cells. It has been reported that Paneth cell-specific deletion of Wnt3 leads to a reduction in small intestinal tumors but not colonic tumors in Apc^Min/+^ mice [43]. We speculated that Wnt3 production might be reduced in the LAT1^fl/fl^; vil-cre small intestine, and as expected, Wnt3 expression was substantially reduced at both the mRNA and protein levels (Fig. 6e, f). To test the possible involvement of two other Wnts, Wnt6 and Wnt9b, which are shown to be expressed in the intestinal epithelium [44], we first compared the expression levels of the three Wnts in the tissue. As expected, Wnt3 showed the strongest expression; Wnt6 and Wnt9b levels were not affected by LAT1 deletion (Fig. S9). These data suggest that Wnt3 is the major Wnt affecting tumor initiation in our setting. We also evaluated the alteration in Wnt/β-catenin pathway activation by LAT1 deficiency in vitro; the expression of Wnt/β-catenin target genes, *Ccnd1* and *Axin2*, was significantly reduced. *c-Myc* also showed a trend of reduced expression in LAT1^fl/fl^; vil-cre; Apc^Min/+^-derived organoids compared with that in the LAT1^fl/fl^; Apc^Min/+^ -derived organoids. This indicated that Wnt/β-catenin pathway activation was indeed inhibited by LAT1 deficiency (Fig. 6g). Finally, we confirmed that spherical formation was recovered in LAT1^fl/fl^; vil-cre; Apc^Min/+^-derived organoids by supplementing the medium with Wnt3; however, the diameter was smaller than that of LAT1^fl/fl^; Apc^Min/+^-derived organoids. This is in accordance with our results suggesting that the role of LAT1 in cell proliferation is dependent on the mTORC1 pathway (Fig. 6h, i). These data indicate that LAT1 expression in the small intestinal crypts plays a critical role in tumor development partly through Wnt3 production, presumably mainly from Paneth cells affecting the activation of the Wnt/β-catenin pathway. However, once a tumor develops, the mTORC1 pathway downstream of LAT1 supports the acceleration of tumor growth.

## Discussion

LAT1 is generally described to be absent in the gastrointestinal normal epithelium, although it is upregulated in many cancers [14]. Surprisingly, we found that LAT1 is constitutively expressed in the normal crypt base, where actively proliferating cells, including intestinal stem cells reside. Our data suggest that abundant amino acid availability may be involved in the risk of intestinal tumor initiation. Further, once a tumor is initiated, LAT1-positive cells may have a growth advantage and become larger than LAT1-negative cells.

The mTORC1 pathway is a master regulator of cell growth and a major sensor of amino acids in the environment [24, 45]. Although the intestinal epithelium constantly proliferates, mTORC1 was dispensable to maintain a normal intestine but required for tumor development in Apc^Min/+^ mice [30]. Our findings are consistent with the previous study, as no gross defects, such as those in the birth ratio or growth, were detected in LAT1^fl/fl^; vil-cre mice. Nevertheless, the observation of fewer Paneth cells should not be ignored since Paneth cells provide antimicrobial peptides and components of stem cell niches, such as Wnt3. LAT1 is dispensable in normal tissue development; however, it remains possible that the LAT1 plays a critical role in a non-steady-state situation, such as intestinal infection and/or tissue damage. It can be readily supposed that a reduction in Paneth cell number may expose the host to higher susceptibility to intestinal infection or possibly delay intestinal tissue repair when damaged. The function of LAT1 in maintaining the health of the non-tumor tissues requires further investigation.

Herein, LAT1 deficiency played a role in tumor initiation by reduction of Wnt3. However, how exactly the initiation was blocked remains to be investigated, along with how a reduction in Paneth cell number occurred in LAT1^fl/fl^; vil-cre mice. It is possible that LAT1 is expressed in Paneth cells, and its deletion could have directly affected Paneth cell development. Alternatively, LAT1 may be expressed in stem cells, and deletion of LAT1 could have affected stem cell differentiation to Paneth cells, or both. Apc^Min/+^ tumor is initiated via loss of heterozygosity. LAT1 may affect DNA stability and play a role in inducing the loss of heterozygosity [46]; however, these speculations should be confirmed in future studies.

The stress of insufficient nutrients, such as amino acids, can trigger autophagy to supply the required metabolic substrates to fulfill bioenergetic needs. It can also result in apoptosis when the cell cannot adapt to the insufficient nutrient environment [47]. Our results showed that apoptosis was promoted in LAT1^fl/fl^; vil-cre; Apc^Min/+^mice in vivo but was not recapitulated in the organoid study in vitro. Increased expression of *DDIT3* was confirmed, suggesting that ER stress may have been triggered in vivo*.* Increased expression of ER stress response-related genes has also been reported in Apc^fl/fl^; Kras^G12D/+^; Slc7a5^fl/fl^; Villin^CreER^ mice compared with that in Apc^fl/fl^; Kras^G12D/+^; Slc7a5^+/+^; Villin^CreER^ mice [48]. These results indicated that ER stress induction was not due to mTOR inhibition alone because mTOR blockage with rapamycin did not induce these changes [48]. However, the activation of autophagy was detected in the absence of LAT1 [48]. Autophagy could have been activated in the tumors of LAT1^fl/fl^; vil-cre; Apc^Min/+^ mice, but it was not adequate to support tumor cell energy demands, triggering ER stress and apoptosis. Alternatively, LAT1 deficiency could have caused impaired autophagy, failing to generate energy to adapt to the environment, leading to the increased apoptosis observed with the promoted cleavage of Caspase-3 in vivo (Fig. 5d, e). It would be worth investigating the precise mechanism by which apoptosis was triggered in the absence of LAT1.

Finally, we did not observe any difference in colonic tumors in our experimental setting. It would be acceptable to explain that the tumor number was not affected because the colon lacks Paneth cells; our data is further supported by a previous report, which showed Paneth cell-specific deletion of *Wnt3* blocked Apc^Min/+^ tumor formation in the small intestine but not in the colon [43]. Even so, LAT1 deficiency could have influenced tumor size. We found that the amino acid concentration in the colonic contents was extremely low compared with that in small intestinal contents and serum levels of amino acids were similar between LAT1^fl/fl^ and LAT1^fl/fl^; vil-cre mice (data not shown). However, as LAT1 is expressed at the basolateral side of cells [49], the low amino acid levels in the colonic contents do not explain our observations. Thus, the mechanisms underlying tumor development in the small intestines and the colon may be distinct. Nevertheless, our results should not exclude the possibility that LAT1 plays a role in colonic tumor development; we reasoned that if the diet contained a higher number of amino acids, it may have supplied more amino acids into circulation, and/or the colon could provide a growth advantage to LAT1-expressing colonic tumors.

In conclusion, our findings showed that LAT1 is expressed in intestinal crypt cells. Conditional deletion of LAT1 resulted in a reduced number of Paneth cells and decreased Wnt3 production while suppressing tumor development and promoting apoptosis, which benefited Apc^Min/+^ mice with a small number of small-sized tumors. Our findings indicate that the nutrient environment and the fate of intestinal tumors may be connected, at least via amino acid transporter LAT1 expression. Further investigations may help elucidate the link between nutrient intake and intestinal tumor development and may shed light on scientifically evidenced prophylactic measures to avoid intestinal cancer development in terms of a daily diet.

## Supplementary Information

Below is the link to the electronic supplementary material.

Supplementary file1 (PDF 1191 kb)
