# Correction: LAT1 expression influences Paneth cell number and tumor development in Apc^Min/+^ mice

**DOI:** 10.1007/s00535-023-01975-y

**Published:** 2023-03-08

**Authors:** Yunlong Sui, Namiko Hoshi, Ryuichi Ohgaki, Lingling Kong, Ryutaro Yoshida, Norihiro Okamoto, Masato Kinoshita, Haruka Miyazaki, Yuna Ku, Eri Tokunaga, Yuki Ito, Daisuke Watanabe, Makoto Ooi, Masakazu Shinohara, Kengo Sasaki, Yoh Zen, Takenori Kotani, Takashi Matozaki, Zibin Tian, Yoshikatsu Kanai, Yuzo Kodama

**Affiliations:** 1 Division of Gastroenterology, Department of Internal Medicine, Kobe University Graduate School of Medicine, Hyogo, 650-0017 Japan; 2 Department of Bio-system Pharmacology, Graduate School of Medicine, Osaka University, Osaka, 565-0871 Japan; 3 Integrated Frontier Research for Medical Science Division, Institute for Open and Transdisciplinary Research Initiatives (OTRI), Osaka University, Osaka, 565-0871 Japan; 4 Division of Molecular Epidemiology, Kobe University Graduate School of Medicine, Hyogo, 650-0017 Japan; 5 The Integrated Center for Mass Spectrometry, Kobe University Graduate School of Medicine, Hyogo, 650-0017 Japan; 6 Graduate School of Science, Technology and Innovation, Kobe University, Hyogo, 657-8501 Japan; 7 Institute of Liver Studies, King’s College Hospital, London, SE5 9RS UK; 8 Division of Molecular and Cellular Signaling, Department of Biochemistry and Molecular Biology, Kobe University Graduate School of Medicine, Hyogo, 650-0017 Japan; 9 Department of Gastroenterology, The Affiliated Hospital of Qingdao University, Qingdao, 266000 China

**Correction: J Gastroenterol** 10.1007/s00535-023-01960-5

In the original publication of the article, Fig. 6 was published without panels h and i erroneously.

The original article has been corrected.

