# Supplementary material for: LAT1 expression influences Paneth cell number and tumor development in ApcMin/+ mice
Source: J Gastroenterol. 2023 Feb 5;58(5):444–57. doi: 10.1007/s00535-023-01960-5 (PMC10140238; doi:10.1007/s00535-023-01960-5)
Supplement: Supplementary file 1 — Supplementary file1 (PDF 1191 kb) [file 535_2023_1960_MOESM1_ESM.pdf]

Supplementary Information

LAT1 expression influences Paneth cell number and tumor development in $Apc^{Min/+}$ mice

Yunlong Sui[1], Namiko Hoshi[1*], Ryuichi Ohgaki[2,3], Lingling Kong[1], Ryutaro Yoshida[1], Norihiro Okamoto[1], Masato Kinoshita[1], Haruka Miyazaki[1], Yuna Ku[1], Eri Tokunaga[1], Yuki Ito[1], Daisuke Watanabe[1], Makoto Ooi[1], Masakazu Shinohara[4,5], Kengo Sasaki[6], Yoh Zen[7], Takenori Kotani[8], Takashi Matozaki[8], Zibin Tian[9], Yoshikatsu Kanai[2,3], Yuzo Kodama[1]

[1] Division of Gastroenterology, Department of Internal Medicine, Kobe University Graduate School of Medicine, Hyogo 650-0017, Japan

[2] Department of Bio-system Pharmacology, Graduate School of Medicine, Osaka University, Osaka 565-0871, Japan

[3] Integrated Frontier Research for Medical Science Division, Institute for Open and Transdisciplinary Research Initiatives (OTRI), Osaka University, Osaka 565-0871, Japan

[4] Division of Molecular Epidemiology, Kobe University Graduate School of Medicine, Hyogo 650-0017, Japan

[5] The Integrated Center for Mass Spectrometry, Kobe University Graduate School of Medicine, Hyogo 650-0017, Japan

[6] Graduate School of Science, Technology and Innovation, Kobe University, Hyogo 657-8501, Japan

[7] Institute of Liver Studies, King's College Hospital, London SE5 9RS, United Kingdom

[8] Division of Molecular and Cellular Signaling, Department of Biochemistry and Molecular Biology, Kobe University Graduate School of Medicine, Hyogo 650-0017, Japan

[9] Department of Gastroenterology, the Affiliated Hospital of Qingdao University, Qingdao, 266000, China

[*]**Corresponding author:** Namiko Hoshi. Postal address: 650-0017**.** Email: nhoshi@med.kobe-u.ac.jp**.** Phone number: +81-78-382-5774

Supplementary methods

Histological analysis

Colonic specimens harvested from patients who underwent colonoscopy at Kobe University Hospital between January 1995 and December 2020 were used. This retrospective observational analysis was approved by the ethics committee of Kobe University Hospital (approval number B210022). Tissues were paraffin embedded, sectioned, and subjected to standard immunohistochemistry techniques. A primary antibody for LAT1 (1:500; transgenic; #KE023) and secondary antibody (Dako, #K4003) were used. For Olfm4 staining in the mouse crypts, the primary antibody Olfm4 (1: 200; Cell Signaling, #39141) was used.

Western blotting

Whole mouse small intestine tissues, which contain normal and tumor regions, were harvested and homogenized on ice using a Physcotron homogenizer NS-310E II (Microtec) in 3.5 mL/0.1 mg tissue of lysis buffer (20 mM Tris-HCl [pH 7.4], 150 mM NaCl, 1 mM EDTA, and protease inhibitor cocktail [Roche Diagnostics]). After centrifugation at 1,000 × g for 5 min, the supernatants were collected and subjected to ultracentrifugation at 391,000 × g for 1 h to obtain crude membrane pellets. The pellets were suspended in lysis buffer containing 10% (v/v) glycerol, adjusted to a protein concentration of 2 mg/mL, and solubilized with 1% (w/v) Fos-Choline-12 on ice. After removing insoluble materials via centrifugation at 20,400 × g for 15 min, the supernatants were mixed with Laemmli buffer. Proteins were resolved via SDS-PAGE and transferred onto PVDF membranes (GE Healthcare Life Science). Anti-mouse LAT1 chicken antibody (provided by Osaka University) and anti-Na+/K+-ATPase α1 mouse antibody (Santa Cruz Biotechnology, sc-21712) were used as primary antibodies; incubation with the

primary antibodies was followed by incubation with horseradish peroxidase-conjugated secondary antibodies. Chemiluminescent signals were detected using ECL Prime Western Blotting Detection Reagent with Amersham Imager 680 (GE Healthcare), and densitometric analysis was performed using ImageJ (NIH).

DNA extraction from small intestinal contents and analysis of microbiota

DNA was extracted using an automated DNA isolation system (GENE PREP STAR PI-480 KURABO) as previously described (Takahashi S, et al., 2014). The V3-V4 regions of 16S rRNA were amplified. Barcoded amplicons were paired-end sequenced using the MiSeq system (Illumina). The primer sequences on paired-end sequencing reads were trimmed by Cutadapt ver 1.18 with default settings (Martin M, et al., 2011). Paired-end sequencing reads were merged using fastq-join program with default settings (Aronesty E, et al., 2013). Only joined-reads that had quality value scores $\geq$ 20 for more than 99% of the sequence were extracted using FASTX-Toolkit (Gordon A, et al., 2010). The chimeric sequences were deleted with usearch61 (Caporaso JG, et al., 2010, Edgar RC, et al., 2011). Nonchimeric reads were submitted for 16S rDNA-based taxonomic analysis using the Ribosomal Database Project ver 2.13 (RDP) and the TechnoSuruga Lab Microbial Identification database ver 16.0 (DB-BA, TechnoSuruga Laboratory) with homology $\geq$ 97 % (Wang Q, et al., 2007, Kasai C, et al., 2015). Principal coordinate analysis (PCoA) was performed using Rstudio (Inc., Boston), assessed via ANOSIM.

Gene knockdown by RNAi

LoVo and SW480 cells were maintained at 37 °C with 5% CO_2/95% air in an RPMI-1640 medium supplemented with 10%(v/v) heat-inactivated FCS. One day after the passage in 6-cm

dishes (2.5 × 10^5 cells/dish), cells were transfected with Silencer Select siRNA of LAT1 (s15655) or Negative Control #2 (Ambion) using Lipofectamine RNAiMAX (Invitrogen), cultured for two days, further incubated in RPMI-1640 medium supplemented with 10%(v/v) heat-inactivated FCS containing various concentrations of amino acids (1×, 0.25×, and 0.125× of normal concentration) for 24 h, and collected for western blotting. Anti-caspase-3 mouse antibody (Santa Cruz Biotechnology, sc-271028) and anti-LAT1 rabbit antibody (TransGenic, KE026) were used as primary antibodies for western blotting.

RNA scope

Mouse small intestines were harvested and paraffin-embedded. RNAScope was performed using a Multiplex Fluorescent Reagent Kit v2 according to the manufacturer's instructions (Advanced Cell Diagnostic, #323100-USM). The probe Lgr5 (1:50; Advanced Cell Diagnostic, #312171-C2) was used.

Measurement of amino acid levels in the plasma via LC-MS/MS

At 15 weeks of age, blood samples were collected from the mice. To quantify plasma amino acid levels, 10 μL of the internal amino acid standard mixture (APDSTAG, FUJIFILM-WAKO, #293-73701) was added to 10 μL of plasma. After 1 h of incubation with 1 mL of methanol on ice, the samples were centrifuged, and the supernatant was subjected to LC-MS/MS. The system consisted of a Q-Trap 6500 (Sciex) equipped with a Shimadzu LC-30AD HPLC system. For amino acid analysis, an Intrada Amino Acid column (100 mm × 3.0 mm, 3.0 μm, Imtakt Co) was used with an acetonitrile/formic acid/100 mM ammonium formate gradient of 100:0.1:0 to 0:0:100 (v/v/v) at a 0.6 mL/min flow rate. For monitoring and quantifying the

levels of amino acids, the multiple reaction monitoring (MRM) method was developed with signature ion pairs Q1 (parent ion)/Q3 (characteristic fragment ion) for each molecule.

Supplementary Figures

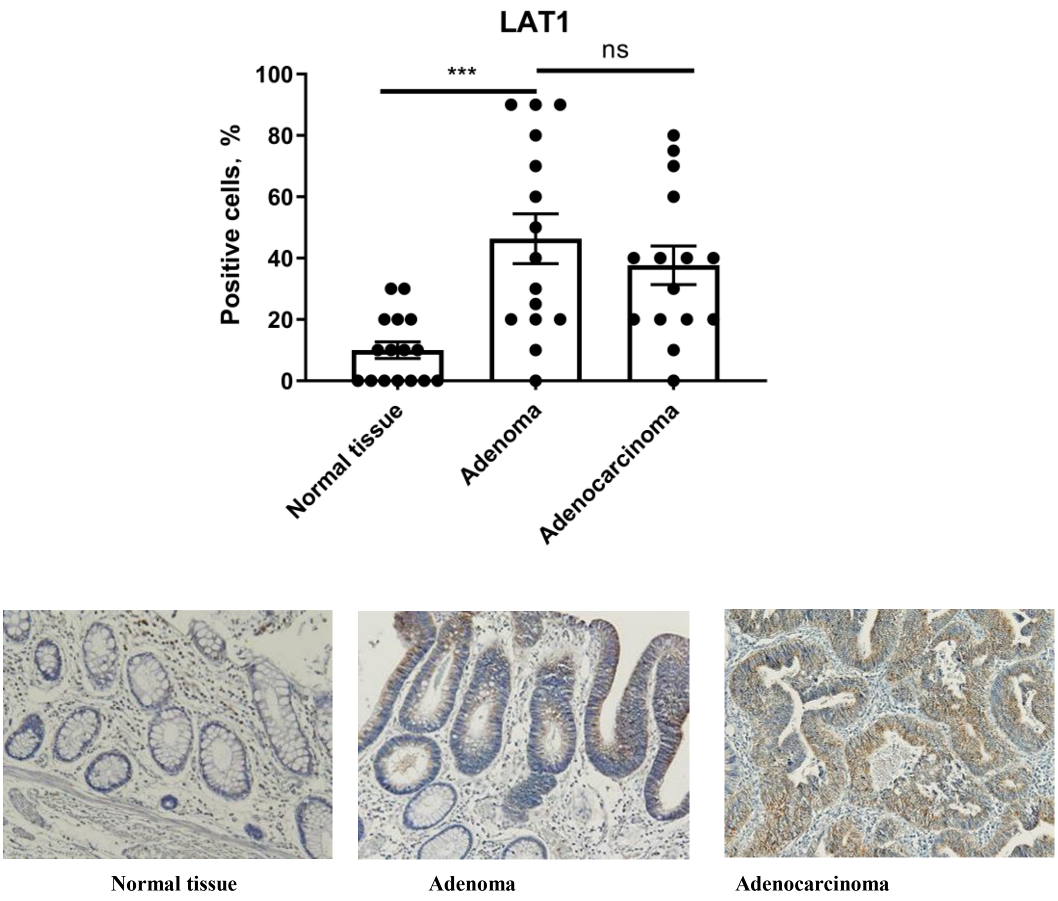

Fig. S1 LAT1 expression in human tissues, as demonstrated via immunohistochemical staining. The following tissues were harvested from the human colon: normal tissues (n = 16), adenoma tissues (n = 15), and adenocarcinoma tissues (n = 15). Magnification, 200×. Error bars indicate the mean ± standard error of the mean (SEM); statistical analysis was performed using one-way ANOVA followed by Bonferroni's multiple-comparisons test. $^{***}p < 0.001$. ns: no significance, $p > 0.05$.

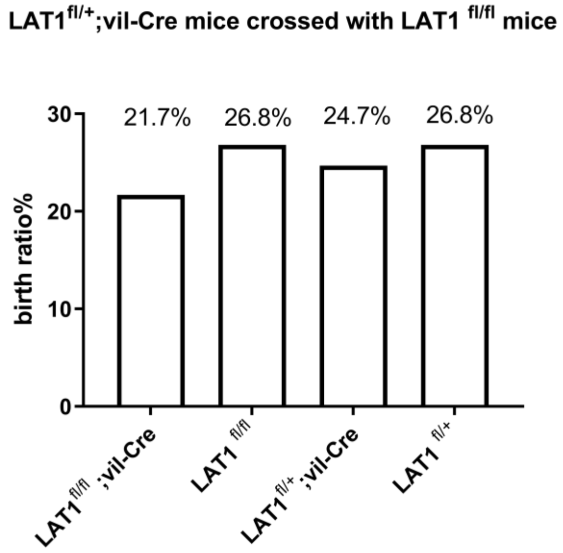

Fig. S2 Conditional deletion of LAT1 does not affect the birth ratio. LAT1$^{fl/+}$; vil-cre mice were crossed with LAT1$^{fl/fl}$ mice, and the birth ratio of offspring was calculated. In total, 97 mice were subjected to analysis: LAT1$^{fl/fl}$;vil-cre mice 21.7% (n = 21), LAT1$^{fl/fl}$ mice 26.8% (n = 26), LAT1$^{fl/+}$;vil-cre mice 24.7% (n = 24), and LAT1$^{fl/+}$ mice 26.8% (n = 26).

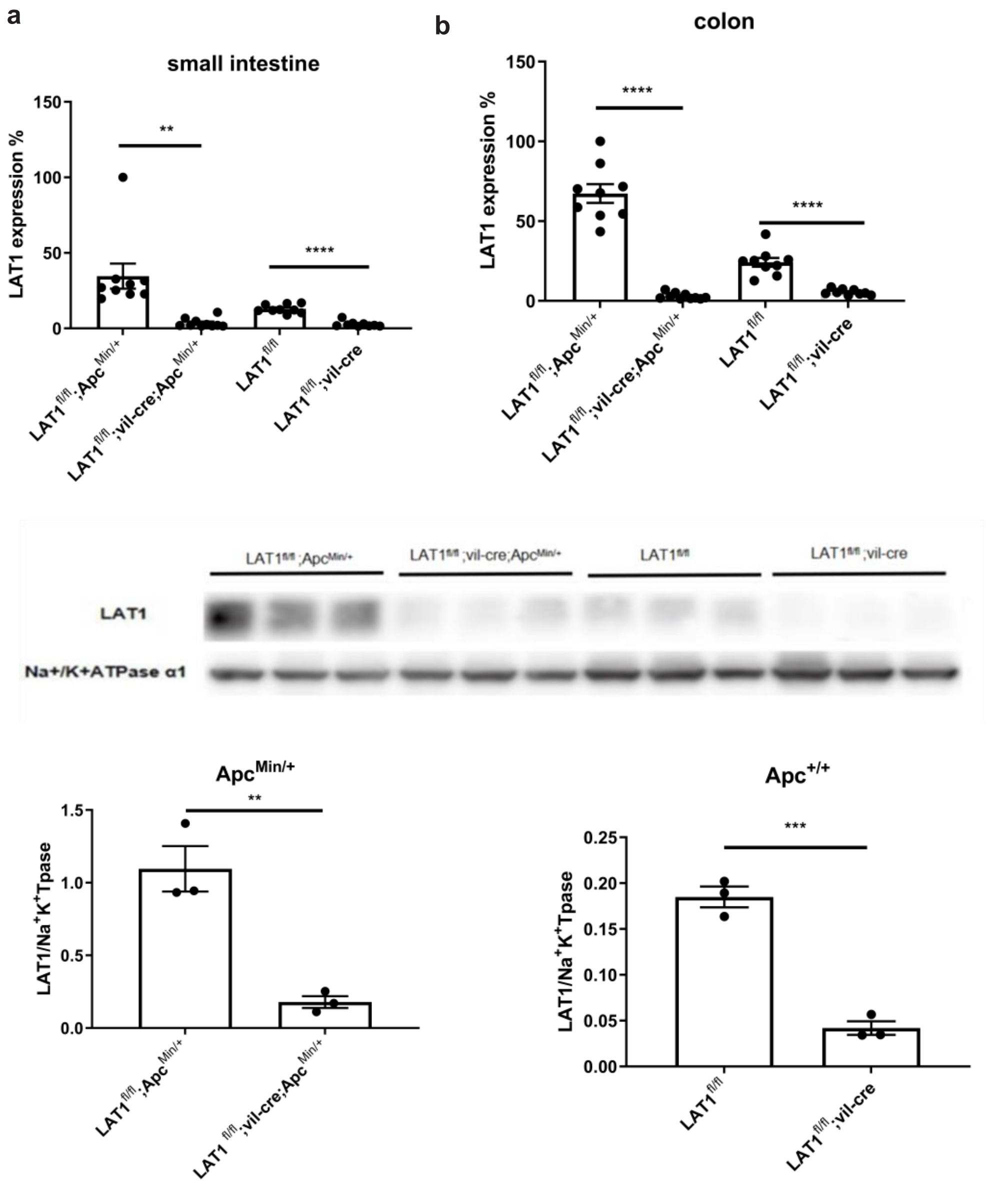

Fig. S3 LAT1 deletion efficiency. The expression of LAT1 in the (**a**) small intestine and (**b**) colon was analyzed via real-time PCR. Normal tissues were harvested from LAT1$^{fl/fl}$ (n = 9) and LAT1$^{fl/fl}$; vil-cre mice (n = 9). Tumor tissues were harvested from LAT1$^{fl/fl}$; Apc$^{Min/+}$ (n = 9) and LAT1$^{fl/fl}$; vil-cre; Apc$^{Min/+}$ mice (n = 10). (**c**) Western blots for LAT1 in the small intestines from each group are shown. Error bars indicate the mean ± SEM; statistical analysis was performed using an unpaired two-tailed Student's *t*-test. $^{**}p < 0.01$, $^{***}p < 0.001$, $^{****}p < 0.0001$.

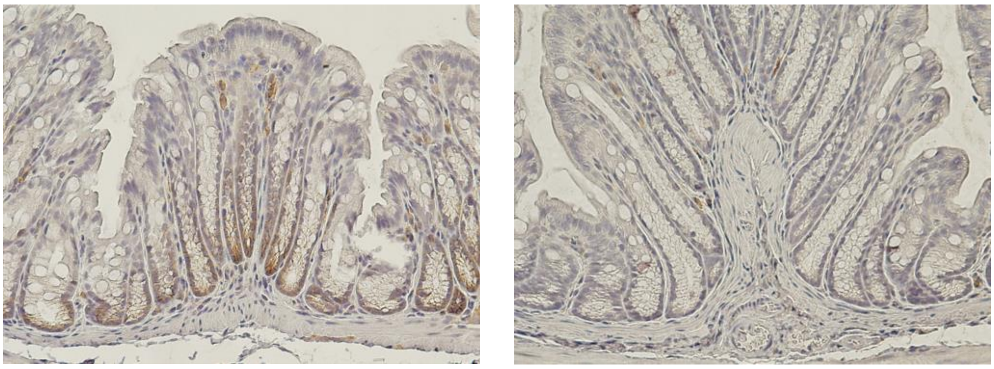

LAT1$^{fl/fl}$ **LAT1$^{fl/fl}$;vil-cre**

Fig. S4 LAT1 is expressed at the crypt base in the colon. Tissues were harvested from the mouse colon, and immunohistochemical staining of LAT1 in the colon was performed. The representative pictures are presented (400× magnification).

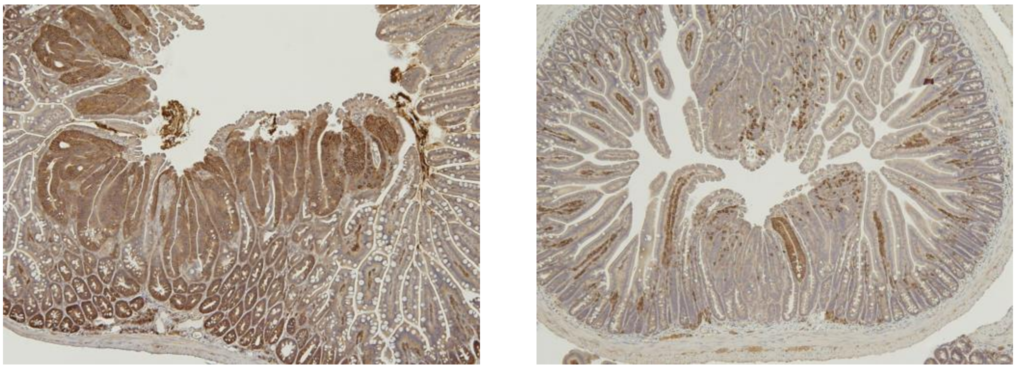

LAT1$^{fl/fl}$;Apc$^{Min/+}$ **LAT1$^{fl/fl}$;vil-cre;Apc$^{Min/+}$**

Fig. S5 The entire tumor shows a high expression of LAT1 in the LAT1-sufficient intestine, but LAT1 deficiency remains associated with tumor development. Tissues, including tumors, were harvested from the mouse small intestine, and immunohistochemical staining of LAT1 was performed. The representative pictures are presented (100× magnification).

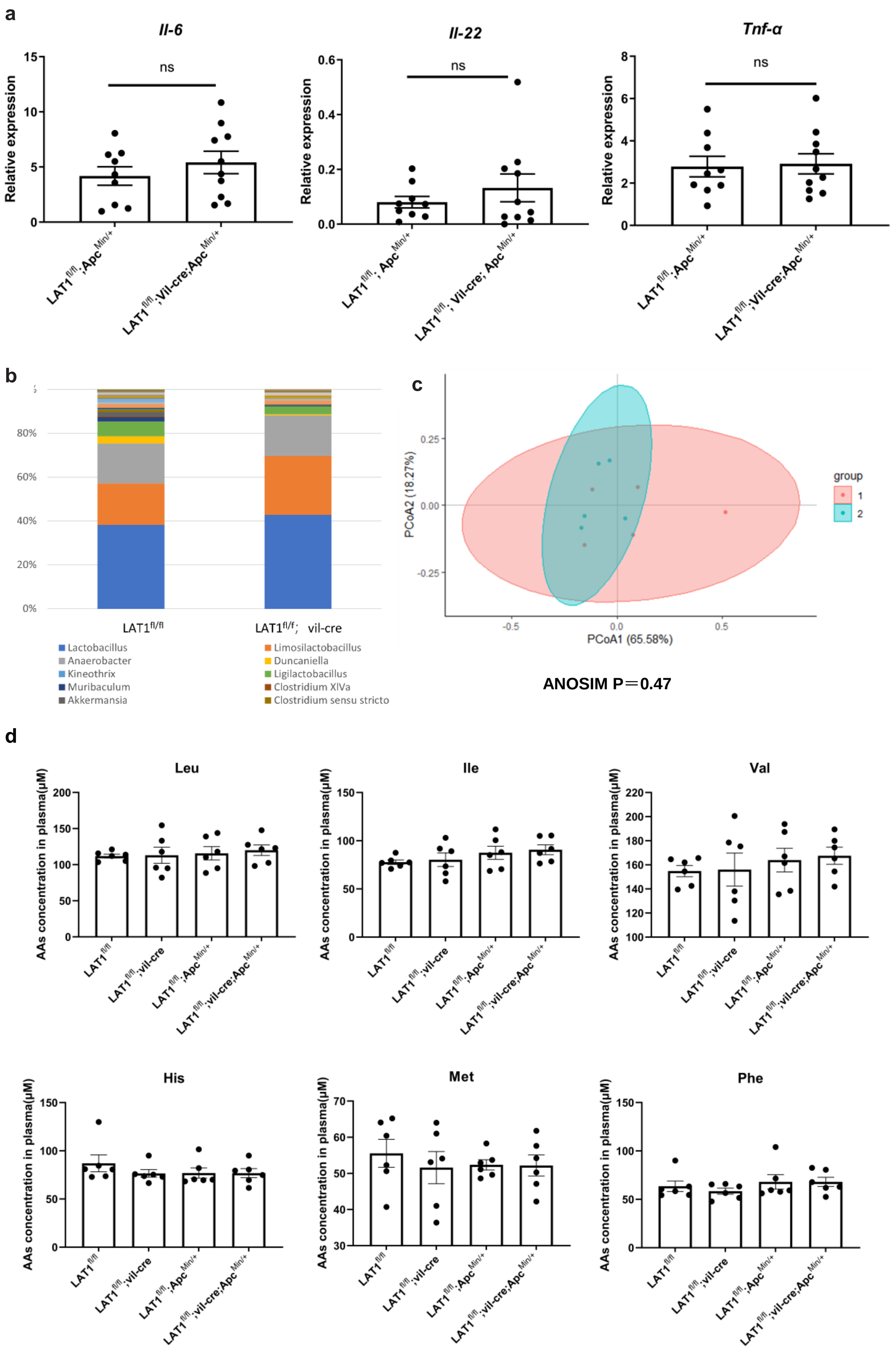
a
Il-6
Il-22
Tnf-α
ns
Relative expression
LAT1 fl/fl ;Apc Min/+
LAT1 fl/fl ;Vil-cre;Apc Min/+
b
80%
60%
40%
20%
0%
LAT1fl/fl
LAT1fl/f; vil-cre
Lactobacillus
Limosilactobacillus
Anaerobacter
Duncaniella
Kineothrix
Ligilactobacillus
Muribaculum
Clostridium XlVa
Akkermansia
Clostridium sensu stricto
c
PCoA2 (18.27%)
PCoA1 (65.58%)
group
1
2
ANOSIM P＝0.47
d
Leu
Ile
Val
His
Met
Phe
AAs concentration in plasma(μM)
LAT1fl/fl
LAT1 fl/fl ;vil-cre
LAT1 fl/fl ;Apc Min/+
LAT1 fl/fl ;vil-cre;Apc Min/+

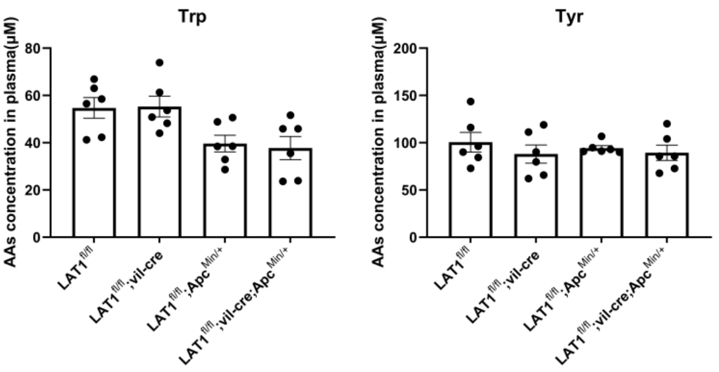

Fig. S6 Deletion of LAT1 does not significantly affect the tissue inflammatory status, gut microbiota, or amino acid levels in the circulation. (**a**) The expression of *Il-6*, *Il-22*, and *Tnf-α* in the tumors was analyzed via real-time PCR. Tumor tissues were harvested from LAT1$^{fl/fl}$; Apc$^{Min/+}$ (n=9) and LAT1$^{fl/fl}$; vil-cre; Apc$^{Min/+}$ mice (n=10). Analysis of the intestinal microbiota via 16S rRNA sequencing: (**b**) Microbiota composition at the genus level in the small intestinal contents. The major genus names are indicated under the graph. (**c**). Principal coordinate analysis (PCoA) plot: group 1 (red), LAT1$^{fl/fl}$ mice (n = 5); group 2 (light green): LAT1$^{fl/fl}$; vil-cre mice (n = 5). Differences in microbial composition were assessed using ANOSIM. (d). Plasma concentrations of the eight amino acids known to be transported via LAT1 were measured. LAT1$^{fl/fl}$ (n=6), LAT1$^{fl/fl}$; vil-cre (n=6), LAT1$^{fl/fl}$; Apc$^{Min/+}$(n=6) , LAT1$^{fl/fl}$; vil-cre; Apc$^{Min/+}$(n=6). Error bars indicate the mean ± SEM; statistical analysis was performed using an unpaired two-tailed Student's t-test. ns: no significance, $p > 0.05$.

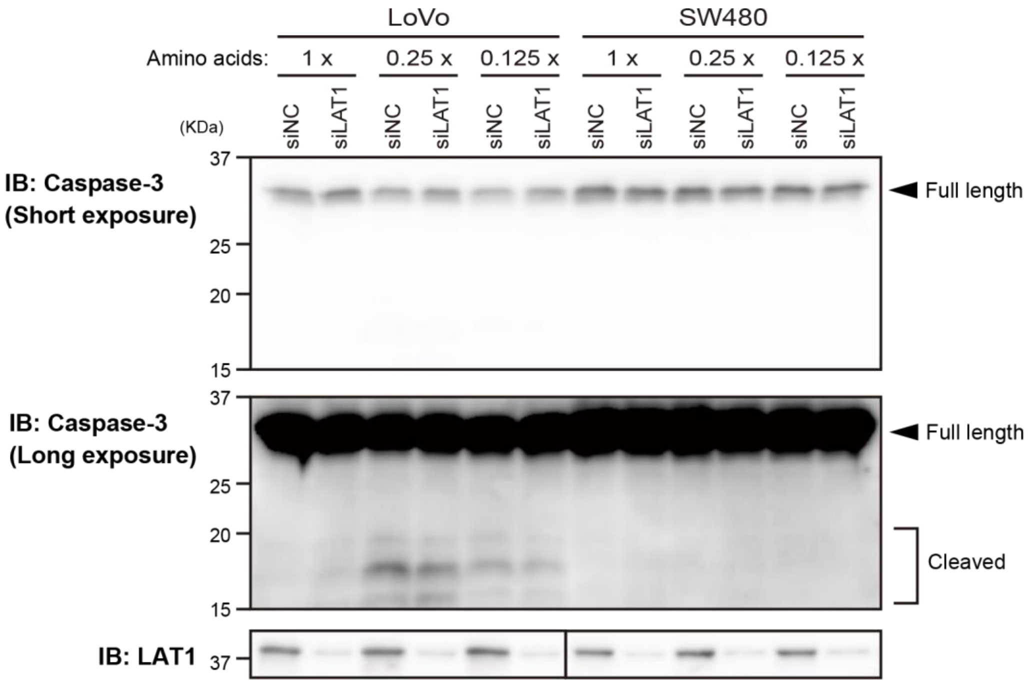

Fig. S7 Knockdown of LAT1 does not increase caspase-3 cleavage. Cleavage of caspase-3 (upper two panels) and expression of LAT1 (bottom panel) were analyzed via western blot in LAT1-knocked-down LoVo and SW480 cells. Cells were transfected with either the negative control siRNA (siNC) or LAT1-targeted siRNA (siLAT1). Results from the cells incubated in RPMI 1640 medium with various amino acid concentrations (1×, 0.25×, and 0.125× of normal concentration) are shown.

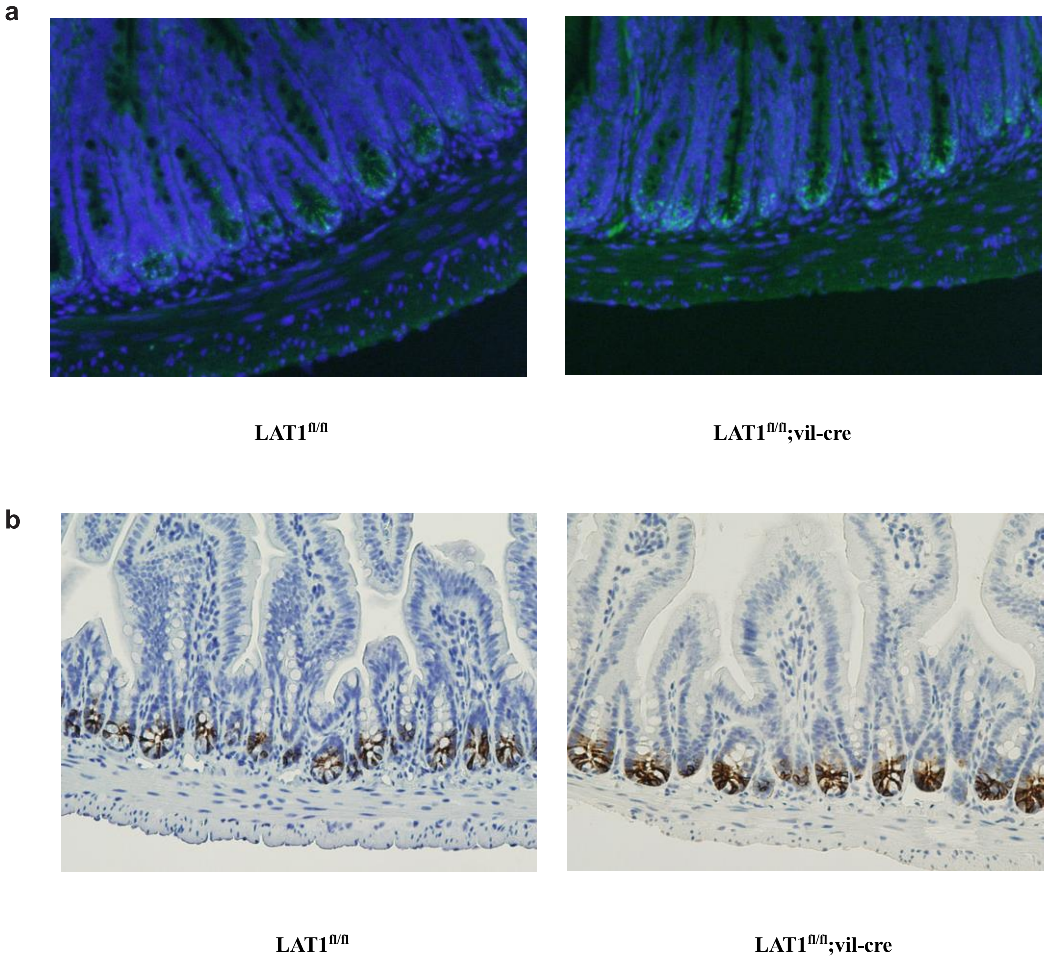

Fig. S8 Deletion of LAT1 in the small intestine may not affect the number of stem cells. Tissues were harvested from mouse small intestines. **(a)** In situ hybridization (RNAscope) of Lgr5 (green) and **(b)** immunohistochemical staining of Olfm4 are shown (400× magnification).

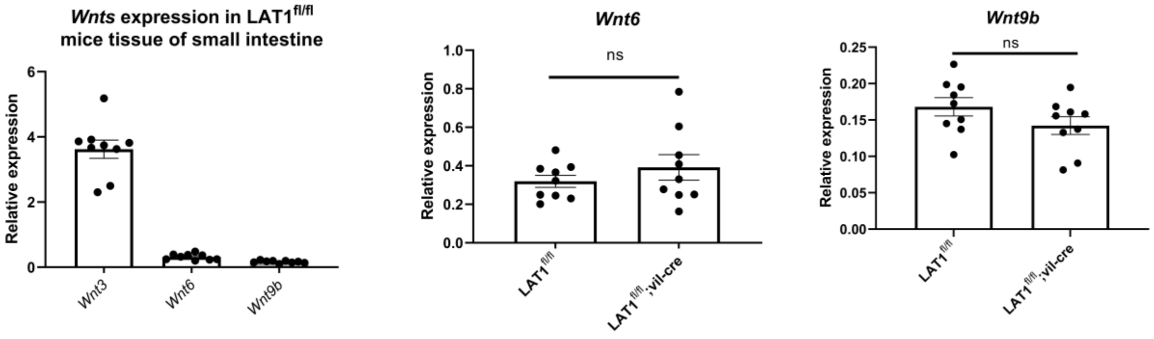

Fig. S9 Comparison of *Wnt* expression levels in the small intestinal tissue

Three Wnt genes known to be expressed in the small intestinal epithelium were analyzed using real-time PCR. *Wnt3* expression was found to be higher than *Wnt6* and *Wnt9b* expression. *Wnt6* and *Wnt9b* expression levels were not affected by the deletion of LAT1. LAT1$^{fl/fl}$ (n=9), LAT1$^{fl/fl}$; vil-cre (n=9). Error bars indicate the mean ± SEM; statistical analysis was performed using an unpaired two-tailed Student's t-test. ns: no significance.
